# Bibliometric Mapping of Studies Assessing Genotoxicity and Cytotoxicity From Cone Beam Computed Tomography Versus Panoramic Radiography Using Exfoliated Buccal Cells

**DOI:** 10.7759/cureus.110578

**Published:** 2026-06-10

**Authors:** Shaik Mohamed Shamsudeen, Smitha Naik

**Affiliations:** 1 Oral and Maxillofacial Pathology and Oral Microbiology, Pacific Academy of Higher Education and Research University, Udaipur, IND

**Keywords:** cbct, cytotoxicity, genotoxicity, opg, radiation

## Abstract

Radiation-induced cellular damage is a well-known phenomenon; its negative effects during diagnostic procedures have received less attention. Numerous diagnostic modalities are being examined for their impact on cells, and interest in this field has been steadily increasing. There is a gap in the bibliometric features of this field of study since the theme evolution, prominent institutions, and journals reporting them have been examined less, and no bibliometric analysis on the cytotoxic and genotoxic effects of dental radiation has been published thus far. The present bibliometric analysis aimed to evaluate the methodological characteristics, geographical distribution, global research trends, citation patterns of studies, and institutional contributions of published literature that investigated the genotoxic and cytotoxic effects of cone beam computed tomography (CBCT) and panoramic radiography on oral mucosal cells. PubMed, Scopus, and Web of Science were searched for original research studies using search strategies designed and customized according to the database. Publications according to year, country, and modality of imaging were first analyzed. Subsequently, the study sample size, the type of analysis used, institutional contributions, journal-wise output, funding sources, radiation dose used, and top-cited papers were reported based on the collected data. Thirty-five articles were included in the bibliometric analysis. There is a gradual increase in publications over time. Orthopantomogram (OPG) is the most common assessing modality, followed by CBCT. Micronucleus assays have been predominantly performed, followed by genotoxicity and cytotoxicity assays. Bibliometric analysis showed a rise in interest in this subject, although geographic distribution and methodology were not satisfactory. Future studies may bear these facts in mind and proceed in a more logical direction, enhancing the knowledge of toxicity due to diagnostic radiation.

## Introduction and background

The advent of radiographic imaging a century ago marked a new era in dental diagnosis and research. It became widely used in practice and the standard for diagnosis in a very short period of time. As time passed, improvements in radiographic science brought a lot of advancements into dental diagnostic radiology [1]. The imaging modalities commonly used today are intraoral X-rays and panoramic (orthopantomogram (OPG)) X-rays. Adding to the arsenal, cone beam computed tomography (CBCT) is now dominating the arena [2].

Every imaging modality has a specific property in tissue visualization, offering an advantage to the clinician. Hence, extensive radiological visualization has been thought to improve the diagnosis and treatment planning for the benefit of the patient. OPG offers a two-dimensional visualization of the jaws and teeth and remains the standard first-line imaging technique in general dental practice. In contrast, CBCT offers three-dimensional visualization with high spatial resolution and is therefore preferred for areas such as implant planning, orthodontic assessments, endodontic evaluations, and maxillofacial surgery [3,4].

It has been known for a long time that ionizing radiation causes cytotoxic effects on tissues and is hence used for radiotherapy at higher doses. Until recently, the diagnostic-level radiation was considered absolutely safe and free from such cytotoxic effects. When studies reported the cytotoxicity of diagnostic imaging, it became a major game-changer in the field of imaging, and the focus shifted towards minimal use of radiology for diagnostic, therapeutic, and prognostic purposes, forming the as low as reasonably achievable (ALARA) principle [5-8].

As time passed, investigations using more sophisticated techniques were introduced into clinical practice, especially in the past couple of decades. To assess the cytotoxic effects due to diagnostic dental radiology, buccal mucosa is often selected as it is directly exposed to X-rays during dental imaging. Also, obtaining the sample is essentially non-invasive. Among the techniques used, cytotoxicity and genotoxicity analyses have been extensively explored, in addition to a few studies reporting immunohistochemistry and other molecular techniques.

Although there is a large amount of published data on the biological effects of dental radiographic procedures, there is a lack of a comprehensive bibliometric assessment of the literature. Hence, it is important to understand geographical distribution, methodological approaches, publication trends, and research gaps to guide future investigations and improve radiation safety research. Since the research topic is recently emerging and is of significant interest in the field of dental radiology, a comprehensive bibliometric evaluation is needed, especially on the global research trends, influential authors, and thematic evolution in this field. This will widen the understanding of how the research on CBCT and OPG-associated genotoxicity has evolved, in addition to highlighting not only influential works but also research gaps and future directions for both research and safe radiologic practice. Therefore, the present study was undertaken to provide a bibliometric overview of published evidence concerning the genotoxic and cytotoxic effects associated with CBCT and panoramic radiography. 1. How many and which studies compare CBCT and OPG for genotoxicity/cytotoxicity in buccal cells?; 2. What are the temporal trends, leading authors/institutions/countries, and top journals?; 3. What are the dominant assays/outcome measures?

## Review

Materials and methods

Study Design

This study aimed to assess the bibliometric characteristics of articles reporting cytotoxic and genotoxic effects of dental X-rays, OPG, and CBCT as observed from exfoliated buccal epithelial cells.

Data Sources

**Figure 1:**
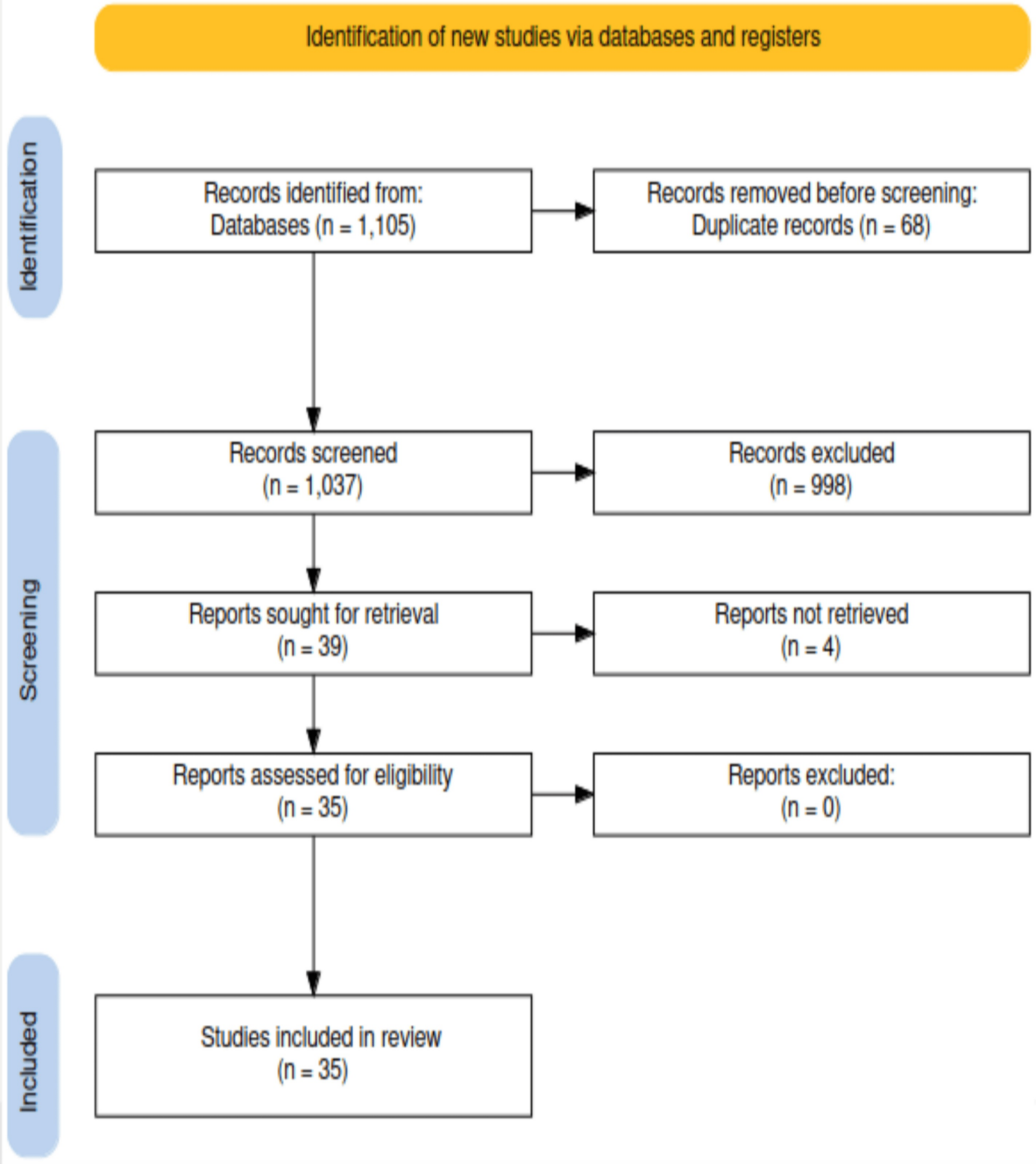
PRISMA flowchart. PRISMA 2020 flow diagram demonstrating the literature search, screening, and selection process. PRISMA: Preferred Reporting Items for Systematic reviews and Meta-Analyses.

Three major scientific databases were included: PubMed (MEDLINE), which contains biomedical and dental science literature; Scopus (Elsevier), which contains multidisciplinary and citation-analysis coverage; and Web of Science (Clarivate Analytics), which has reference and co-citation mapping. The study was conducted up to September 2025, and no restrictions were placed on publication year or language. The present study was conducted by adhering to Preferred Reporting Items for Systematic reviews and Meta-Analyses (PRISMA) 2020 guidelines [9]. Two independent reviewers participated in each stage, including title and abstract screening and article selection. Any disagreement was solved through discussion and consensus. Figure 1 represents the PRISMA flow diagram.

Eligibility Criteria

Only original research studies from peer-reviewed journal articles were included, and items like conference abstracts, editorials, letters, and book chapters were excluded.

Search strategy: Search queries were designed and customized according to the database. The details are as follows: *PubMed Search String* ("Cone-Beam Computed Tomography"[MeSH] OR "cone beam computed tomography"[tiab] OR CBCT[tiab] OR "Radiography, Panoramic"[MeSH] OR panoramic[tiab] OR orthopantomograph*[tiab] OR OPG[tiab]) AND ("Buccal Mucosa"[MeSH] OR "Mouth Mucosa"[MeSH] OR buccal[tiab] OR "oral mucosa"[tiab] OR "exfoliated cells"[tiab] OR "epithelial cells"[tiab]) AND (genotoxic*[tiab] OR cytotoxic*[tiab] OR "micronucleus"[tiab] OR micronuclei[tiab] OR "micronucleus assay"[tiab] OR "nuclear anomalies"[tiab] OR "cytome assay"[tiab] OR "DNA damage"[tiab]).

Scopus search string:* *TITLE-ABS-KEY(("cone beam computed tomography" OR CBCT OR "panoramic radiography" OR panoramic OR orthopantomograph* OR OPG OR OPT)) AND TITLE-ABS-KEY(("buccal mucosa" OR "oral mucosa" OR buccal OR "buccal cells" OR "exfoliated cells" OR "oral epithelial" OR "epithelial cells")) AND TITLE-ABS-KEY((genotoxic* OR cytotoxic* OR "micronucleus" OR micronuclei OR "micronucleus assay" OR "cytome assay" OR "nuclear anomalies" OR "DNA damage")).

Web of Science (WoS) search string: TS=(("cone beam computed tomography" OR CBCT OR "panoramic radiography" OR panoramic OR orthopantomograph* OR OPG OR OPT)) AND TS=(("buccal mucosa" OR "oral mucosa" OR buccal OR "buccal cells" OR "exfoliated cells" OR "oral epithelial" OR "epithelial cells")) AND TS=((genotoxic* OR cytotoxic* OR "micronucleus" OR micronuclei OR "micronucleus assay" OR "cytome assay" OR "nuclear anomalies" OR "DNA damage").

Data Extraction and Management

All records retrieved from the three databases were exported in BibTeX format and imported into RStudio (version 4.3.0, RStudio, Inc., Boston, MA, USA) using the bibliometric package (v4.2.0; 2017). Duplicate entries were removed based on DOI and metadata. The final dataset was consolidated, and data were extracted. The extracted variables included authors, year, journal, country, institution, study design, modality (CBCT/OPG/both), sample size, age group, assay used, exposure parameters, outcome metrics, citations, and funding sources. Each parameter was tabulated, and data were obtained accordingly. The outcome measures were yearly trends, country analysis, institutional output or top 10 institutions, journal distribution--journals vs publication count, modality comparison (CBCT vs OPG), funding agencies, top 10 cited papers, the assay methods used, and exposure parameters summary.

Results

The search results from PubMed (41), Scopus (64), and Web of Science (1000) were consolidated to obtain 1105 articles, and after removing duplicates, 1037 articles remained. After screening for eligibility criteria, 35 articles were included in the bibliometric analysis [10-44] (Table 1).

**Table 1:** Data extraction. Summary of literature data. OPG: orthopantomogram; CBCT: cone beam computed tomography; MDCT: multi-detector computed tomography; IHC: immunohistochemistry; FAPESP: Fundação de Amparo à Pesquisa do Estado de São Paulo (São Paulo Research Foundation); FOV: field of view; CAT: computed axial tomography.

Authors	Year	Journal	Country	Institution	Study design	Modality (CBCT/OPG/both)	Sample size	Age group	Assay used	Exposure parameters	Outcome metrics	Citations	Funding
Anbumeena et al. [10]	2021	J Indian Acad Oral Med Radiol	India	SRM Dental College, Chennai	Cross-sectional	OPG	60	6-60 years	Micronucleus/cytotoxic biomonitoring	65–79 kV/8 mA/12 s	Genotoxic and cytotoxic markers (inferred)	6	Nil
Angelieri et al. [11]	2010	Dentomaxillofac Radiol	Brazil	São Paulo Methodist University UMESP, São Paulo	Mutagenicity/cytotoxicity assessment	Orthodontic radiographs (OPG/other dental radiographs)	18	14.2 ± 1.4 yrs	Micronucleus/cytotoxicity assays	250–71 kV/15 mA/14 s/110 mGy/cm^2^	Mutagenicity and cytotoxicity indices	67	FAPESP
Arora et al. [12]	2014	J Dent (Tehran)	India	Kalka Dental College, Meerut	Micronucleus assay study	OPG	53	25.21 ±12.67	Micronucleus assay	74 kV and 10 mA, 12 s with output dose rate of 0.325 m Gy/s at 70 kV, 10 mA	Micronuclei frequency	43	Nil
Ayres et al. [13]	2023	Diagn Cytopathol	Brazil	Federal University of Sergipe (UFS), Aracaju	Comparative evaluation	CBCT (two CBCT types compared)	18	31 ± 0.5	Mutagenic assays on oral mucosa cells	CS 8100 3D: 84 kVp, 3.2 mA, 15 s of scan time. I-CAT: 120 kVp, 3.6 mA during 40 s of scan time	Mutagenic effects	3	Coordenação de Aperfeiçoa- mento de Pessoal de Nível Superior—Brazil
Bakr et al. [14]	2025	BMC Oral Health	Egypt	Mansoura University, Mansoura	Comparative by age groups	CBCT	40	14-35	Genotoxic and cytotoxic assays	84 kV, 9–14 mA, field of view 80 × 100 mm, and exposure time was 6 s	Genotoxic and cytotoxic markers	0	Nil
Batool et al. [15]	2024	Technol Health Care	Pakistan	Postgraduate Medical Institute, Lahore,	Indicator study	OPG	74	18-40	Micronuclei counts	71–73 kV with 12 mA, 12 s with output dose rate of 0.325 m Gy/s at 72 kV, 12 mA	Micronuclei frequency	1	Deanship of Scientific Research at King Khalid University
Carlin et al. [16]	2010	Dentomaxillofac Radiol	Brazil	Federal University of São Paulo, UNIFESP, SP	Biomonitoring study	CBCT	19	26.8 ± 5	DNA damage and cytotoxicity assays	80 kV, a tube current of 4 mA, and an exposure time of 40 s	DNA damage markers; cytotoxicity	61	FAPESP
Cerqueira et al. [17]	2004	Mutat Res	Brazil	State University of Feira de Santana, Bahia,	Genetic damage assessment	OPG	31	24 ± 1.023	Micronucleus/genotoxic assays	250-71 kV/15 mA/14 s	Genetic damage frequency	129	FAPESP
Cerqueira et al. [18]	2008	Dentomaxillofac Radiol	Brazil	Salvador	Genotoxic effects study	OPG	40	26 ± 9.18	Genotoxic assays	65–90 kV, 15 mA, 14 s, 110 mGy cm^2^, effective dose 21.4 mSv	Genotoxic markers	116	FAPESP and CNPq student fellowship
da Fonte et al. [19]	2018	Dentomaxillofac Radiol	Brazil	Federal University of Sergipe, UFS, Aracaju,	Evidence study on CBCT	CBCT	29	45.8 ± 12.5	Genotoxicity and cytotoxicity assays	70 kV, 10 mA, and 32.4 s were used, under a minute voxel size of 200 µm and FOV of 80 × 37 mm	Genotoxicity and cytotoxicity indicators	30	Nil
da Silva et al. [20]	2007	Mutat Res	Brazil	Federal University of Rio Grande do Sul, Porto Alegre,	Nuclear changes study	OPG	42	18-40	Nuclear alterations assessment	60–85 kV, 14–17 s, 10 mA	Nuclear changes in tongue epithelium	54	Nil
Faeli Ghadikolaei et al. [21]	2023	Caspian J Intern Med	Iran	Babol University of Medical Sciences, Babol	Field of view effects	CBCT	60	34.24 ± 7.76	Genotoxicity and cytotoxicity assays	90 kVp and 8 mA	Genotoxicity and cytotoxicity markers	11	Babol University of Medical Sciences
Jahanshahiafshar et al. [22]	2023	Iran J Med Sci	Iran	Babol University of Medical Sciences, Babol	Comparative CBCT vs MDCT	CBCT and MDCT	60	21-50	Genotoxic and cytotoxic assays	CBCT: 85-90 kVp and 8 mA; MDCT: 110 kVp tube voltage, 35 mA tube current	Comparative genotoxicity/cytotoxicity	6	Babol University of Medical Sciences
Karabas et al. [23]	2019	Niger J Clin Pract	Turkey	Istanbul University, Istanbul	Cell and DNA damage evaluation	OPG	30	20-46	DNA damage assays	-66-74 kV, 5-8 mA, and 13.1-13.9 s	Comet assay	9	Nil
Kaur et al. [24]	2012	World J Dent	India	Maharishi Markandeshwar College of Dental Sciences and Research, Mullana	Genotoxic effects and recovery time evaluation	OPG	100	15-50	Micronucleus assay	Not reported	Micronuclei	4	Nil
Li et al. [25]	2018	Sci Rep	China	Peking University School and Hospital of Stomatology, Beijing	Buccal mucosa damage evaluation	Dental X-ray (general)	98	23.63 ± 6.64	Genotoxicity assays	66 kVp, 4–10 mA, 17.6 s for the panoramic radiographs and 77 kVp, 12 mA, 0.5–1.0 s for the lateral radiographs, 77 kVp, 12 mA, 0.8–1.2 s for the posteroanterior radiographs	Buccal mucosa cell damage	25	Nil
Luke et al. [26]	2021	Res J Pharm Tech	UAE	Ajman University, Ajman	Degenerative nuclear alterations study	X-rays (dental)	116	6-75	Micronucleus assay	Not reported	Genotoxicity markers	5	Ajman University
Madhavan et al. [27]	2012	J Indian Acad Oral Med Radiol	India	Ragas dental college and hospital	Cross-sectional	OPG	35	18-65	Genetic damage assays	70 kV, 10 mA	Genetic damage	17	Nil
Malik et al. [28]	2022	J Indian Acad Oral Med Radiol	India	Teerthanker Mahaveer University, Moradabad	Comparative micronuclei vs AgNORs	OPG	100	15-50	Micronuclei and AgNORs	Not reported	Short-term genotoxic effects	2	Nil
Mosavat et al. [29]	2022	Mutat Res Genet Toxicol Environ Mutagen	Iran	Tehran University of Medical Sciences, Tehran	Cytotoxicity, genotoxicity, and IHC study	CBCT	30	20-50	Genotoxicity and cytotoxicity assays; p53 IHC	s 80 kVp, 4 mA, 17 s	Genotoxicity, cytotoxicity, p53 expression	5	Vice Research Center of Tehran University of Medical Sciences
Mounika et al. [30]	2021	J Indian Acad Oral Med Radiol	India	Lenora Institute of Dental Sciences, Rajahmundry	Genomic damage evaluation	CBCT	60	23-50	Genomic damage assays	Tube voltage of 76 kVp, a tube current of 9 mA, with the exposure time of 8 seconds	Genomic damage	6	Nil
Popova et al. [31]	2007	Dentomaxillofac Radiol	Bulgaria	National Centre of Radiobiology and Radiation Protection, Sofia	Micronucleus test	OPG	32	24-73	Micronucleus test	Not reported	Micronuclei frequency	85	Nil
Ribeiro [32]	2019	Dentomaxillofac Radiol	Brazil	Federal University of Sergipe, UFS, Aracaju,	Evidence study	CBCT	29	45.8 ± 12.5	Genotoxicity and cytotoxicity assays	70 kV, 10 mA, and 32.4 s	Genotoxicity and cytotoxicity	7	Nil
Sandhu et al. [33]	2015	J Indian Acad Oral Med Radiol	India	Kanti Devi Dental College and Hospital, Mathura	Genotoxic effect evaluation	OPG	100	Not reported	Genotoxicity assays	Not reported	Genotoxic effect	17	Nil
Santhosh et al. [34]	2020	J Contemp Dent Pract	India	Karpaga Vinayaga Institute of Dental Sciences, Kanchipuram	Cross-sectional	OPG	30	24-65	Nuclear changes assessment	Not reported	Nuclear changes in buccal mucosa	6	Nil
Sheikh et al. [35]	2012	Quintessence Int	India	Maharishi Markandeshwar College of Dental Sciences and Research, Mullana	Genotoxicity study	OPG	80	Not available	Genotoxicity assays	Not available	Genotoxic markers	21	Nil
Sreeenivas et al. [36]	2014	J Orofac Sci	India	Annoor Dental College & Hospital, Muvattupuzha	Cross-sectional micronuclei assay	CBCT (focus vs scatter)	35	18-60	Micronuclei assay	90 kV, 3 mA, 9 seconds, 11.96 mGy radiation dose,	Localized genotoxicity	1	Kerala State Council for Science, Technology and Environment
Sreeshyla et al. [37]	2023	BBRJ	India	JSS Academy of Higher Education and Research, Mysore	Micronuclei study	OPG	60	10-40	Micronuclei	-	Micronuclei frequency	2	Nil
Thomas et al. [38]	2012	Anal Quant Cytol Histol	India	Annoor Dental College, Kerala	Micronuclei and nuclear anomalies	X-ray radiation (dental)	90	Not available	Micronuclei and nuclear anomalies		MN and nuclear anomalies	10	Nil
Torabinia et al. [39]	2014	Clin Exp Dent Res	Iran	Isfahan University of Medical Sciences, Isfahan	Cross-sectional	OPG	36	18-55	Micronucleus assay	66 kVp, 7.1 mA, and 15.8 s	Micronucleus formation	1	Isfahan University of Medical Sciences
Vishwadha et al. [40]	2024	Cureus	India	SVS Institute of Dental Sciences, Mahabubnagar	Assessing genotoxicity/cytotoxicity at varied FOVs	CBCT	66	16-66	Genotoxicity and cytotoxicity assays	90 kV and 8 mA	Genotoxicity and cytotoxicity	0	Nil
Waingade and Medikeri [41]	2012	Indian J Dent Res	India	Sinhgad Dental College and Hospital, Vadgaon	Micronuclei analysis	OPG	60	12-65	Micronucleus assay	-	Micronuclei frequency	51	Nil
Yang et al. [42]	2017	Dentomaxillofac Radiol	China	Peking University School & Hospital of Stomatology, Beijing	Cytogenetic biomonitoring comparison	CBCT (comparison among sites)	46	23-42	Cytogenetic biomonitoring	110 kVp and 6.24–14.45 mA, 448.15–730.79 mGy cm^2^.	Comparison across buccal, tongue, and gingival cells	32	Nil
Yanuaryska et al. [43]	2021	Brazilian J Oral Sci	Indonesia	Universitas Gadjah Mada, Indonesia	Alteration of mucosa cell maturation	Different radiographic imaging methods	40	25	Mucosa cell maturation patterns	Analog panoramic radiographs: 90 kVp, 8–10 mA, and 20 s or 72 kVp, 10 mA, and 16 s. The CBCT: 85 kVp, 6 mA, and 16.6 s.	Alteration of maturation pattern	3	Faculty of Dentistry, Universitas Gadjah Mada
Yanuaryska et al. [44]	2021	Archives of Orofacial Science	Indonesia	Universitas Gadjah Mada, Indonesia	Viability and DNA damage study	Panoramic X-ray	20	18-25	Viability and DNA damage assays	90 kVp, 8–10 mA, 20 s.	Viability and DNA damage	1	Dana Masyarakat grant, Universitas Gadjah Mada, Indonesia.

Temporal Trends

**Figure 2:**
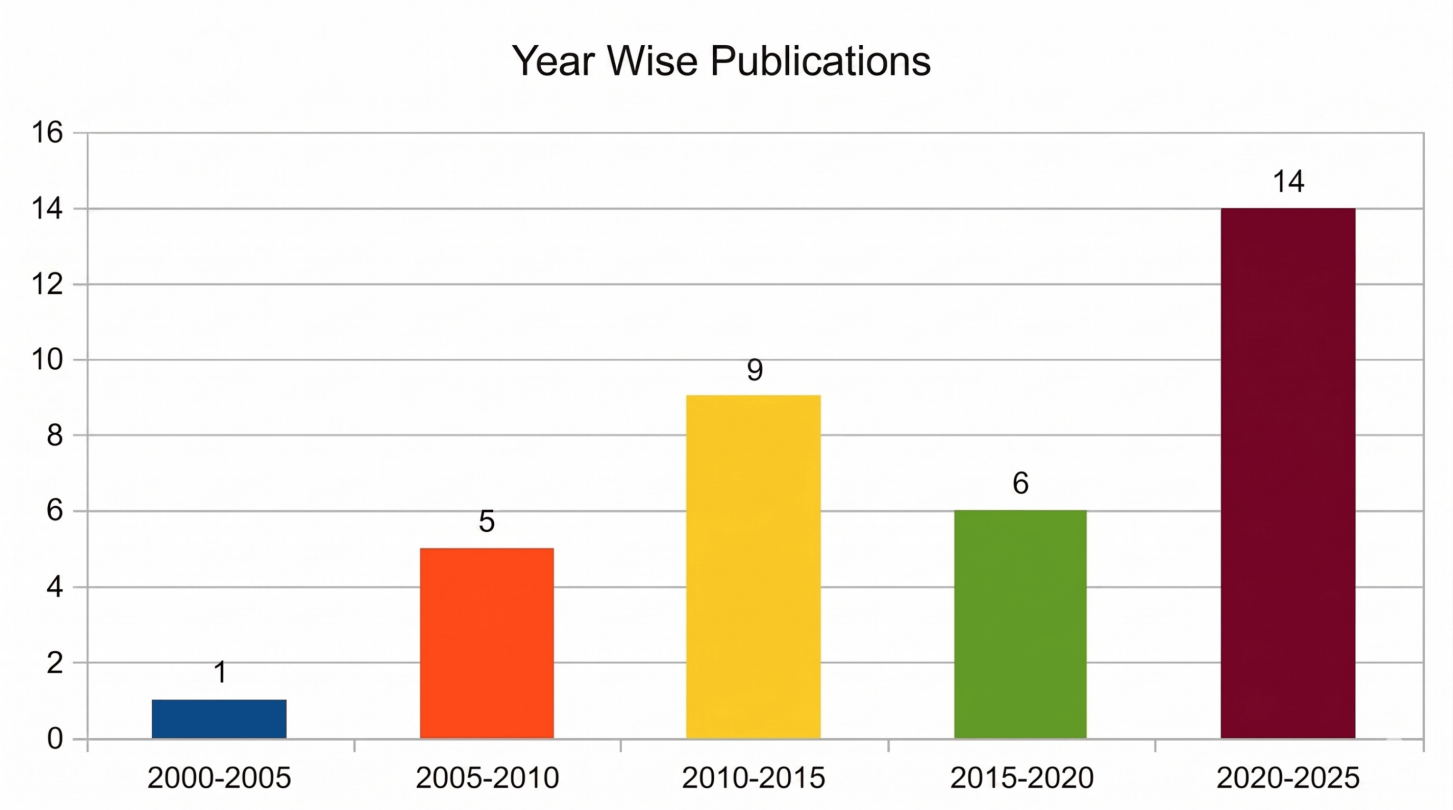
Year-wise distribution of publications.

There is a gradual increase in publications over time. The time period from 2020 to 2025 showed the highest number of publications with 14 articles, suggesting a spike in the interest towards the topic in recent years (Figure 2).

Geographical Distribution

**Figure 3:**
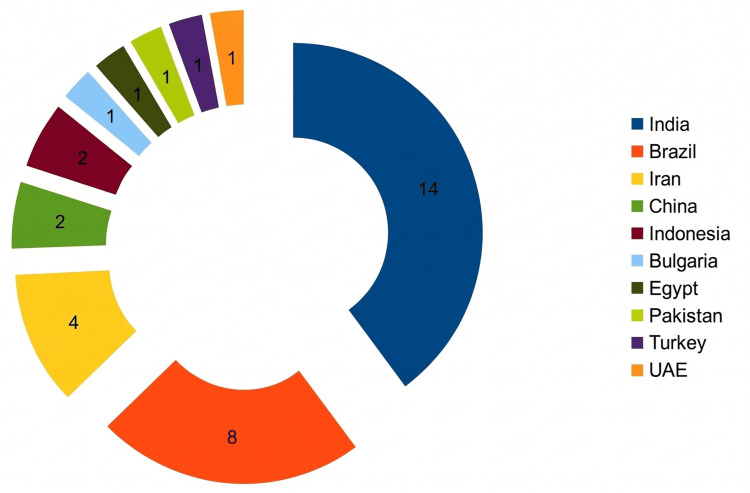
Country-wise articles. Distribution of publications by country of origin.

The country-wise distribution of published work shows that most papers are given by India, Brazil, Iran, and China (Figure 3). The geographic trend shows that research activity on this topic is more prevalent in Asian and South American countries.

Methodological Characteristics

**Figure 4:**
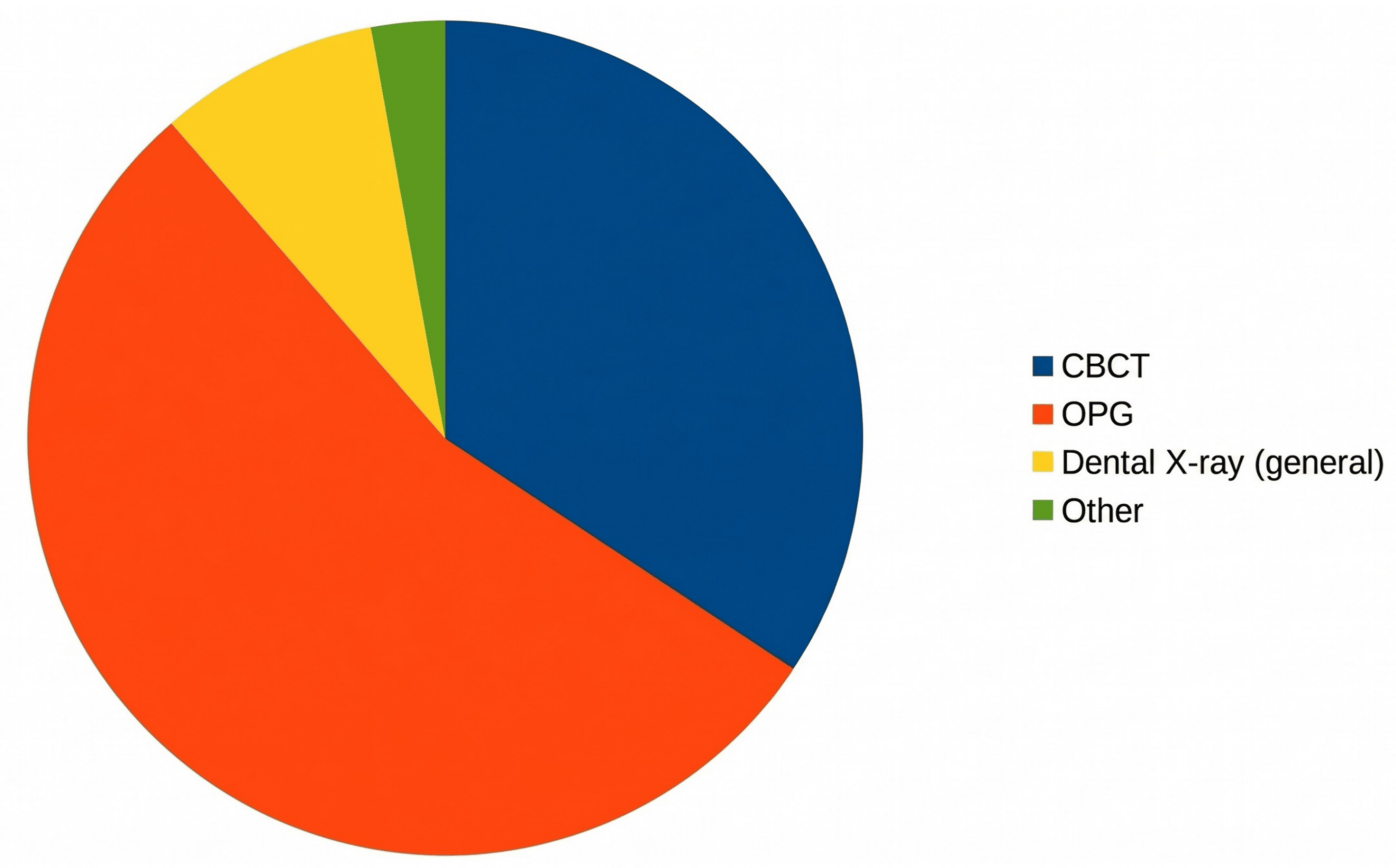
Modality of imaging. Distribution of studies based on the dental imaging techniques used. OPG: orthopantomogram; CBCT: cone beam computed tomography.

Our analysis reveals that OPG is the most frequently evaluated modality, followed by CBCT (Figure 4). Furthermore, there is a clear interest in investigating the cytotoxicity and genotoxicity associated with both radiographic techniques.

**Figure 5:**
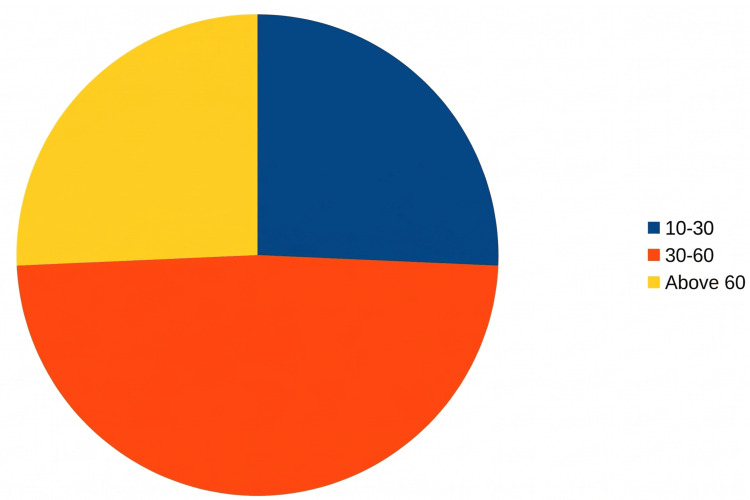
Sample size. Distribution of the study population by number of samples.

Most of the studies limited their sample sizes to below 60. It can be inferred in the future that sample sizes in this interval may be sufficient for the said analyses (Figure 5).

Type of Analysis

Most of the studies concentrated on genotoxic and cytotoxic assays, with a smaller number of studies focusing on other relevant assays. Micronucleus assays have been predominantly performed, followed by genotoxicity and cytotoxicity assays. From the analysis, CBCT studies were more associated with DNA damage and cell viability assessments. On the other hand, OPG studies focused on micronuclei and nuclear anomalies (Table 2).

**Table 2:** Types of analysis used. Summary of biological assay methods, their purposes, and representative literature in dental radiography research. OPG: orthopantomogram; CBCT: cone beam computed tomography.

Assay method	Purpose/biological endpoint	Representative studies (author, year)	Modality	Approx. no. of studies
Micronucleus (MN) assay	Detects chromosomal breakage or loss; biomarker of genotoxicity in exfoliated buccal mucosa cells	Cerqueira et al. (2004, 2008) [17,18]; Kaur et al. (2012) [24]; Arora et al. (2014) [12]; Torabinia et al. (2014) [39]; Batool et al. (2024) [15]; Sreeshyla et al. (2023) [37]; Waingade and Medikeri (2012) [41]; Popova et al. (2007) [31]; Thomas et al. (2012) [38]	Mostly OPG	~13
Cytotoxicity/cell viability assays	Measures cell death, nuclear abnormalities (pyknosis, karyorrhexis, karyolysis) after exposure	Cerqueira et al. (2008) [18]; Carlin et al. (2010) [16]; Angelieri et al. (2010) [11]; Faeli Ghadikolaei et al. (2023) [21]; da Fonte et al. (2018) [19]; Jahanshahiafshar et al. (2023) [22]; Vishwadha et al. (2024) [40]	CBCT and OPG	~8
Genotoxicity assays (general/DNA damage)	Detects DNA strand breaks, oxidative damage, or other genotoxic effects	Li et al. (2018) [25]; Mosavat et al. (2022) [29]; Mounika et al. (2021) [30]; Da Silva et al. (2007) [20]; Anbumeena et al. (2021) [10]; Vishwadha et al. (2024) [40]; Sandhu et al. (2015) [33]; Sheikh et al. (2012) [35]	Both	~10
Comet assay (single cell gel electrophoresis)	Evaluates DNA strand breaks at individual cell level	Karabas et al. (2019) [23]	OPG	1
Nuclear alterations/anomalies assessment	Detects cytological changes (karyorrhexis, karyolysis, binucleation) in epithelial cells	Da Silva et al. (2007) [20]; Santhosh et al. (2020) [34]; Thomas et al. (2012) [38]; Luke et al. (2021) [26]	OPG/dental X-ray	~4
AgNOR staining	Evaluates nucleolar organizer regions; marker of cellular proliferation and genotoxic stress	Malik et al. (2022) [28]	OPG	1
Cytogenetic biomonitoring	Compares cytogenetic responses among different oral sites (buccal, gingival, tongue)	Yang et al. (2017) [42]	CBCT	1
Genomic damage/biomonitoring assays (combined MN + nuclear abnormalities)	Comprehensive evaluation of nuclear aberrations and MN together	Angelieri et al. (2010) [11]; Carlin et al. (2010) [16]; Mounika et al. (2021) [30]; Bakr et al. (2025) [14]	OPG and CBCT	~5
p53 Immunohistochemistry (IHC)	Protein-level biomarker of DNA damage response/apoptosis	Mosavat et al. (2022) [29]	CBCT	1
Mucosa cell maturation/viability and DNA damage assays	Evaluates cellular differentiation patterns and viability post-exposure	Yanuaryska et al. (2021a, 2021b) [43,44]	OPG/CBCT	2

Institutional Contributions

Articles were from diverse institutions, with the maximum papers from the Federal University of Sergipe (UFS) (Table 3). Other institutions had a single study each to their credit, proving a broad and uneven global distribution.

**Table 3:** Institutional contributions. Distribution of publications by contributing academic and medical institutions.

Institutional output	Number
Federal University of Sergipe (UFS), Aracaju	3
Babol University of Medical Sciences, Babol	2
Maharishi Markandeshwar College of Dental Sciences and Research, Mullana	2
Peking University	2
Universitas Gadjah Mada, Indonesia	2
Ajman University, Ajman	1
Annoor Dental College, Kerala	1
Federal University of Rio Grande do Sul, Porto Alegre	1
Federal University of São Paulo, UNIFESP, SP	1
Isfahan University of Medical Sciences, Isfahan	1
Istanbul University, Istanbul	1
Jagadguru Sri Shivarathreeshwara (JSS) Academy of Higher Education and Research, Mysore	1
Kalka Dental College, Meerut	1
Kanti Devi Dental College and Hospital, Mathura	1
Karpaga Vinayaga Institute of Dental Sciences, Kanchipuram	1
Lenora Institute of Dental Sciences, Rajahmundry	1
Mansoura University, Mansoura	1
National Centre of Radiobiology and Radiation Protection, Sofia	1
Annoor Dental College & Hospital, Puthuppady, Muvattupuzha	1
Postgraduate Medical Institute, Lahore	1
Ragas Dental College and Hospital	1
Salvador	1
São Paulo Methodist University UMESP, São Paulo	1
Sinhgad Dental College and Hospital, Vadgaon	1
SRM Dental College, Chennai	1
State University of Feira de Santana, Bahia	1
SVS Institute of Dental Sciences, Mahabubnagar	1
Teerthanker Mahaveer University, Moradabad	1
Tehran University of Medical Sciences, Tehran	1

Journal Contributions

The highest papers were published by Dentomaxillofac Radiol, J Indian Acad Oral Med Radiol, and Mutat Res. Other studies were distributed among 22 journals, clearly showing that the topic is of interest in both general dental and specialized radiology journals (Table 4). Most of the funding was done by São Paulo Research Foundation in Brazil. It can be seen that this funding source has funded 4 studies in this research area.

**Table 4:** Journal-wise output. Distribution of included studies across various academic journals.

Journal	Number
Dentomaxillofac Radiol	7
J Indian Acad Oral Med Radiol	5
Mutat Res	2
Anal Quant Cytol Histol	1
Archives of Orofacial Science	1
BBRJ	1
BMC Oral Health	1
Brazilian J Oral Sci	1
Caspian J Intern Med	1
Clin Exp Dent Res	1
Cureus	1
Diagn Cytopathol	1
Indian J Dent Res	1
Iran J Med Sci	1
J Contemp Dent Pract	1
J Dent (Tehran)	1
J Orofac Sci	1
Mutat Res Genet Toxicol Environ Mutagen	1
Niger J Clin Pract	1
Quintessence Int	1
Res J Pharm Tech	1
Sci Rep	1
Technol Health Care	1
World J Dent	1

Radiation Dosage

In most studies reporting on OPG, the radiation exposure parameters were not reported, probably due to standardized exposure, limiting the analysis capability. However, for CBCT, most of the papers have reported the dosage, and it is well within the acceptable ranges. Therefore, any toxicity reported would be relevant and clinically applicable (Table 5).

**Table 5:** Summary of radiation dosage. Comparison of technical exposure parameters and data reporting for OPG and CBCT imaging. OPG: orthopantomogram; CBCT: cone beam computed tomography; FOV: field of view.

Parameter	OPG range/mean	CBCT range/mean	Remarks
kV/kVp	60–90 (avg ≈73 kV)	70–120 (avg ≈ 86 kV)	CBCT typically uses higher voltage for volumetric imaging
mA	5–15 mA (avg ≈10 mA)	3–14 mA (avg ≈ 8 mA)	CBCT uses a lower current due to pulsed emission
Exposure time	12–17 s (avg ≈14 s)	6–40 s (avg ≈ 18 s)	CBCT varies widely depending on voxel size and FOV
Data availability	~60% of OPG studies report full parameters	~80% of CBCT studies report parameters	OPG data often missing or partially stated

Paper Citations

Top-cited papers have reached above 100 citations, and a spike in interest in the field is far more evident in this regard. These papers were published from 2001 to 2010. Papers published from 2010 to 2020 did reach a good number of citations, and recent publications are still in the queue (Table 6). This means citation is rising with the age of the article, meaning that articles are regularly searched and cited in this field.

**Table 6:** Top-cited papers. Summarizes the top 10 articles by citation volume, identifying the primary authors, publication years, and total citations.

Top 10 cited articles	Year	Citations
Cerqueira et al. [17]	2004	129
Cerqueira et al. [18]	2008	116
Popova et al. [31]	2007	85
Angelieri et al. [11]	2010	67
Carlin et al. [16]	2010	61
da Silva et al. [20]	2007	54
Waingade and Medikeri [41]	2012	51
Arora et al. [12]	2014	43
Yang et al. [42]	2017	32
da Fonte et al. [19]	2018	30

Discussion

The present bibliometric analysis attempted to determine the trend in articles that assessed the genotoxic and cytotoxic effects due to exposure to diagnostic radiation in dentistry. The analysis has clearly shown that there is a progressive rise in research output, particularly in the past years (2020-2025) while two decades ago, the research output showed limited publications. This clearly demonstrates that the interest is emerging over the period of twenty years. It should be noted that there was a lacuna in technology in both imaging and techniques that determine the cytotoxicity and genotoxicity. A plateau between 2010 and 2020 may suggest initial consolidation of research methods succeeded by a spike in the 2020-2025 time period. It can be related to the popularization of CBCT technology and the rise in awareness of radiation safety.

India has emerged as the highest contributor, clearly showcasing the substantial investment in dental research infrastructure and the wide availability of dental imaging studies in clinical practice. Brazil and Iran also showed significant research activity, as seen in the number of published manuscripts in the field. This suggests a regional interest in radiation biomonitoring. Similarly, institutional output analysis has shown concentration of expertise in the Federal University of Sergipe and Babol University. Nevertheless, the large number of single-article institutions talk against this ideology, globalizing the interest but in a fragmented manner.

In radiation dosage, substantial variation was observed among the studies due to variations in equipment specifications and reporting quality. Studies that assessed the adverse effects of OPG reported values ranging between 60-90 kV with 10 mA and exposure times of 12-17 seconds. However, for CBCT, investigators reported 70-120 kVp, lower tube currents, and widely varying scan times, which depended on voxel size and field of view. Also, around 60% of OPG and 80% of CBCT studies provided complete exposure data, which limited the direct comparison. These inconsistencies in reporting are a major limitation.

Considering the site of sampling, most of the included studies collected exfoliated epithelial cells from the buccal mucosa. It is recommended as it is the pathway of direct radiation exposure, and also a reliable substrate for genotoxicity assays. Few studies also sampled the gingival, labial, and tongue mucosa to compare cytogenetic responses among oral tissues with different keratinization levels. Buccal mucosa sampling was still preferable due to its non-invasive nature, high cell turnover, and sensitivity to DNA damage.

Predominantly, OPG was studied (n=19), and this can be attributed to its wide use and availability at a lower cost, facilitating population-level cytogenetic studies. However, in CBCT studies, although there are fewer, they have reported methodology in a precise manner. This has facilitated better correlation between exposure and biological outcomes. The analysis of cells was done mainly for micronucleus assays for genotoxicity assessment. It is a well-established methodology and widely reported. Few papers have used cytotoxicity assays, DNA damage assays, and p53 immunohistochemistry, showing the beginning of a new era in analytical techniques. It can be expected that in the future, more studies may use these methods for reporting toxicity in better ways.

It is an important observation that most studies utilized a small to moderate sample size, with most of the studies using 30-60 as their sample size. This may be due to a multitude of reasons. Availability of samples and expenditure involved in having a higher sample size may have been the most prominent reasons, since studies that are funded were also using smaller sample sizes. This calls for a need to standardize the protocol in future studies.

At this stage, implications for future research must be discussed. This bibliographic analysis has highlighted several lacunae in the subject. All human ethnicities have not been included due to geographic heterogeneity. Newer and advanced techniques of cell damage analysis need to be performed for better results. Future studies will have to consider these factors and tailor the methods to address these limitations.

## Conclusions

From the bibliometric analysis, it can be observed that there is a constant rise in interest regarding toxicity due to diagnostic radiation. The geographic distribution is not all-inclusive, and the methodology may be slightly outdated. Newer and advanced methodologies need to be incorporated. Future studies should address these identified research gaps through standardized methodologies, larger sample sizes, and multicenter collaborations.

## References

[1] Shah N, Bansal N, Logani A (2014). Recent advances in imaging technologies in dentistry. World J Radiol.

[2] Venkatesh E, Elluru SV (2017). Cone beam computed tomography: basics and applications in dentistry. J Istanb Univ Fac Dent.

[3] Jaju PP, Jaju SP (2014). Clinical utility of dental cone-beam computed tomography: current perspectives. Clin Cosmet Investig Dent.

[4] Gupta S, Patil N, Solanki J, Singh R, Laller S (2015). Oral implant imaging: a review. Malays J Med Sci.

[5] Lin EC (2010). Radiation risk from medical imaging. Mayo Clin Proc.

[6] Lumniczky K, Impens N, Armengol G (2021). Low dose ionizing radiation effects on the immune system. Environ Int.

[7] Hong JY, Han K, Jung JH, Kim JS (2019). Association of exposure to diagnostic low-dose ionizing radiation with risk of cancer among youths in South Korea. JAMA Netw Open.

[8] Kitahara CM, Linet MS, Rajaraman P, Ntowe E, Berrington de González A (2015). A new era of low-dose radiation epidemiology. Curr Environ Health Rep.

[9] Page MJ, McKenzie JE, Bossuyt MP (2021). The PRISMA 2020 statement: an updated guideline for reporting systematic reviews. BMJ.

[10] Anbumeena S, Kannan A, Krithika CL, Vasanthi V (2021). Genotoxic and cytotoxic biomonitoring in patients exposed to panoramic dental radiography: comparison between five different age groups. J Indian Acad Oral Med Radiol.

[11] Angelieri F, Carlin V, Saez DM, Pozzi R, Ribeiro DA (2010). Mutagenicity and cytotoxicity assessment in patients undergoing orthodontic radiographs. Dentomaxillofac Radiol.

[12] Arora P, Devi P, Wazir SS (2014). Evaluation of genotoxicity in patients subjected to panoramic radiography by micronucleus assay on epithelial cells of the oral mucosa. J Dent (Tehran).

[13] Ayres LC, Dos Santos MA, da Mota Santana LA (2023). Comparative evaluation of mutagenic effects of two cone-beam computed tomography in oral mucosa cells. Diagn Cytopathol.

[14] Bakr M, Ata F, Elmahdy AS, Mowafey B (2025). Genotoxic and cytotoxic effects of cone beam computed tomography on exfoliated epithelial cells in different age groups. BMC Oral Health.

[15] Batool SA, Chaudhry S, Munir N (2024). Micronuclei as an indicator of genotoxic change in epithelial cells of buccal mucosa after panoramic radiographs. Technol Health Care.

[16] Carlin V, Artioli AJ, Matsumoto MA, Filho HN, Borgo E, Oshima CT, Ribeiro DA (2010). Biomonitoring of DNA damage and cytotoxicity in individuals exposed to cone beam computed tomography. Dentomaxillofac Radiol.

[17] Cerqueira EM, Gomes-Filho IS, Trindade S, Lopes MA, Passos JS, Machado-Santelli GM (2004). Genetic damage in exfoliated cells from oral mucosa of individuals exposed to X-rays during panoramic dental radiographies. Mutat Res.

[18] Cerqueira EM, Meireles JR, Lopes MA, Junqueira VC, Gomes-Filho IS, Trindade S, Machado-Santelli GM (2008). Genotoxic effects of X-rays on keratinized mucosa cells during panoramic dental radiography. Dentomaxillofac Radiol.

[19] da Fonte JB, Andrade TM, Albuquerque RL Jr, de Melo MF, Takeshita WM (2018). Evidence of genotoxicity and cytotoxicity of X-rays in the oral mucosa epithelium of adults subjected to cone beam CT. Dentomaxillofac Radiol.

[20] da Silva AE, Rados PV, da Silva Lauxen I, Gedoz L, Villarinho EA, Fontanella V (2007). Nuclear changes in tongue epithelial cells following panoramic radiography. Mutat Res.

[21] Faeli Ghadikolaei R, Ghorbani H, Seyedmajidi M, Ebrahimnejad Gorji K, Moudi E, Seyedmajidi S (2023). Genotoxicity and cytotoxicity effects of x-rays on the oral mucosa epithelium at different fields of view: a cone beam computed tomography technique. Caspian J Intern Med.

[22] Jahanshahiafshar Z, Ghorbani H, Seyedmajidi M, Nabahati M, Ebrahimnejad Gorji K, Seyedmajidi S, Moudi E (2023). Genotoxic and cytotoxic effects of cone beam computed tomography and multidetector computed tomography on exfoliated buccal epithelial cells. Iran J Med Sci.

[23] Karabas HC, Ozcan I, Sener LT, Guler SD, Albeniz I, Erdem TL (2019). Evaluation of cell and DNA damage induced by panoramic radiography. Niger J Clin Pract.

[24] Kaur I, Sheikh S, Pallagati S, Gupta D, Singh R, Aggarwal A, Mago J (2012). Evaluation of genotoxic effects of panoramic dental radiography on cells of oral mucosa by micronucleus assay and evaluation of time period required by cells of oral mucosa to recover from the genotoxic effects. World J Dent.

[25] Li G, Yang P, Hao S, Hu W, Liang C, Zou BS, Ma XC (2018). Buccal mucosa cell damage in individuals following dental X-ray examinations. Sci Rep.

[26] Luke AM, Khair AM, Kudrutullah S, Mathew S, Fanas SA, Shetty KP, Patnaik R (2021). Evaluation of genotoxicity in buccal mucosa of patients subjected to X-rays by degenerative nuclear alterations study. Res J Pharm Technol.

[27] Madhavan R, Kumaraswamy M, Kailasam S, Kumar SM (2012). Genetic damage in exfoliated cells from oral mucosa of individuals exposed to X-rays after panoramic radiograph: a cross-sectional study. J Indian Acad Oral Med Radiol.

[28] Malik SD, Malik U, Pillai J, Sharma S, Singh M, Lehri S (2012). Comparison between micronuclei and AgNORs in assessing the short-term genotoxic effects of panoramic radiography on oral mucosa: a cross-sectional study. J Indian Acad Oral Med Radiol.

[29] Mosavat F, Mahdavi N, Safari S (2022). Cytotoxicity, genotoxicity, and immunohistochemical expression of p53 in the oral mucosal epithelium of adults following cone-beam computed tomography. Mutat Res Genet Toxicol Environ Mutagen.

[30] Mounika G, Sridevi K, Krishnaveni B, Kumar NP, Naidu H, Sahi BK (2021). Evaluation of genomic damage from buccal epithelial cells in patients subjected to cone beam computed tomography. J Indian Acad Oral Med Radiol.

[31] Popova L, Kishkilova D, Hadjidekova VB (2007). Micronucleus test in buccal epithelium cells from patients subjected to panoramic radiography. Dentomaxillofac Radiol.

[32] Ribeiro D (2019). Evidence of genotoxicity and cytotoxicity of X-rays in the oral mucosa epithelium of adults subjected to cone beam computed tomography. Dentomaxillofac Radiol.

[33] Sandhu M, Mohan V, Kumar JS (2015). Evaluation of genotoxic effect of X-rays on oral mucosa during panoramic radiography. J Indian Acad Oral Med Radiol.

[34] Santhosh K, Manzoor S, Sushanth A, Seralathan S, Rajasekar V, Jacob A (2020). A cross-sectional study to evaluate nuclear changes in buccal mucosa following panoramic radiography. J Contemp Dent Pract.

[35] Sheikh S, Pallagatti S, Grewal H, Kalucha A, Kaur H (2012). Genotoxicity of digital panoramic radiography on oral epithelial tissues. Quintessence Int.

[36] Sreeenivas R, Nadil A, Thomas P, Baby GG, Mathew DG, Latti P, George J (2024). Comparative evaluation of localized genotoxicity between the field of focus and scatter following exposure to cone beam computed tomography using buccal mucosal micronuclei assay: a cross-sectional study. J Orofac Sci.

[37] Sreeshyla HS, Aishwarya R, Hegde U (2023). A study on micronuclei in exfoliated buccal epithelial smears to detect epithelial changes in patients undergoing panoramic radiography. Biomed Biotechnol Res J.

[38] Thomas P, Ramani P, Premkumar P, Natesan A, Sherlin HJ, Chandrasekar T (2012). Micronuclei and other nuclear anomalies in buccal mucosa following exposure to X-ray radiation. Anal Quant Cytol Histol.

[39] Torabinia N, Mehdizadeh M, Keshani F, Mehdizadeh M, Soltani P, Spagnuolo G (2024). Genotoxicity and micronucleus formation as a result of panoramic radiography in epithelial cells of the buccal mucosa: a cross-sectional study in adults. Clin Exp Dent Res.

[40] Vishwadha C, Thanmai JV, Ramlal G, Goud S, Katne T, Reddy PV (2024). Assessing genotoxicity and cytotoxicity induced by X-ray exposure from cone beam computed tomography at varied fields of view. Cureus.

[41] Waingade M, Medikeri RS (2012). Analysis of micronuclei in buccal epithelial cells in patients subjected to panoramic radiography. Indian J Dent Res.

[42] Yang P, Hao S, Gong X, Li G (2017). Cytogenetic biomonitoring in individuals exposed to cone beam CT: comparison among exfoliated buccal mucosa cells, cells of tongue and epithelial gingival cells. Dentomaxillofac Radiol.

[43] Yanuaryska RD, Anggraeni SC, Puspitasari AH, Shantiningsih RR, Mudjosemedi M, Gracea RS (2021). Alteration of mucosa cell maturation pattern after exposure to different radiographic imaging methods. Braz J Oral Sci.

[44] Yanuaryska RD, Anggraeni SC, Puspitasari AH, Shantiningsih RR (2021). Viability and DNA damage of buccal mucosa cells in patients exposed to panoramic X-ray. Arch Orofac Sci.

